# Medical Toxicology Consultations and Mortality Among Patients With Poisonings in the PICU

**DOI:** 10.1001/jamanetworkopen.2024.62139

**Published:** 2025-02-28

**Authors:** Paul M. Wax, Rachel E. Culbreth, Jeffrey Brent, Christina Hantsch, Theresa Mikhailov, Nancy Brundage, Kim Aldy

**Affiliations:** 1 American College of Medical Toxicology, Phoenix, Arizona; 2 Department of Emergency Medicine, Division of Medical Toxicology, University of Texas Southwestern Medical Center, Dallas; 3 Department of Medicine, Division of Pulmonary and Critical Care Medicine, University of Colorado School of Medicine, Aurora; 4 Toxicology Associates, Littleton, Colorado; 5 Department of Pediatrics, Division of Critical Care Medicine, Medical College of Wisconsin, Milwaukee; 6 Virtual Pediatric Systems, Los Angeles, California; 7 Department of Emergency Medicine, Baylor University Medical Center, Dallas, Texas

## Abstract

**Importance:**

Poisonings are a leading cause of preventable mortality and morbidity among children. Pediatric poisonings are commonly managed in pediatric intensive care units (PICUs) and may require complex treatment and specialized evaluation. Physicians specializing in medical toxicology are trained to treat poisoned patients of all ages; however, they are not universally part of the medical staff across hospital systems.

**Objective:**

To determine if there is a difference in mortality and length of stay for patients with toxicological exposures who receive a medical toxicology consultation.

**Design, Setting, and Participants:**

This is a secondary analysis of an ongoing, cross-sectional registry of patients admitted to PICUs from January 2019 to June 2023 as part of the Virtual Pediatric System (VPS), a database of PICU admissions across 135 medical sites in the US. Patients with toxicological exposure who were admitted to PICUs that participate in the VPS database were included. Data analysis occurred from May to August 2024.

**Exposures:**

Medical toxicology consultations.

**Main Outcomes and Measures:**

The primary outcome was mortality in the PICU and in the hospital overall. Secondary outcomes were length of stay in the PICU and in the hospital. Multilevel models were used to assess associations of toxicology consultations with the primary and secondary outcomes.

**Results:**

There were a total of 52 836 patients with toxicological exposures (29 867 aged 12 to <21 years [56.5%]; 29 401 female [55.6%]), including 2857 (5.4%) patients with medical toxicology consultations and 49 979 (94.6%) without a consultation. Patients who received medical toxicology consultations had similar risk of mortality at baseline, as measured by the Pediatric Risk of Mortality, compared with those without consultations. After adjusting for severity of illness, poisoning type, and other covariates, medical toxicology consultations were associated with 64% lower odds of death in the PICU (adjusted odds ratio, 0.36; 95% CI, 0.20 to 0.63) and 61% lower odds of mortality at any time during the hospitalization (aOR, 0.39; 95% CI, 0.24 to 0.64). Additionally, medical toxicology consultations were associated with a 15% reduced PICU length of stay (log estimate, −0.16; 95% CI, −0.21 to −0.11) and 10% reduced hospital length of stay (log estimate, −0.10; 95% CI, −0.14 to −0.06) after adjusting for all covariates and differences across hospital sites.

**Conclusions and Relevance:**

In this study of patients with toxicological exposures requiring PICU care, having a medical toxicology consultation was associated with lower mortality and shorter LOS compared with not having a consultation, which suggests that medical toxicology consultations may provide life-saving treatment, particularly for the most severe poisonings in the PICU.

## Key Points

**Question:**

Are medical toxicology consultations associated with mortality for patients admitted to the pediatric intensive care unit with toxicological exposures?

**Findings:**

This cross-sectional study of 52 836 patients admitted to the pediatric intensive care unit with toxicological exposures found that, after adjusting for severity of illness and other covariates, medical toxicology consultations were associated with significantly lower odds of death in the pediatric intensive care unit at any time during the hospitalization.

**Meaning:**

These findings suggest that medical toxicology consultations may provide life-saving treatment, particularly for the most severe poisonings in the PICU.

## Teaser

This cross-sectional study assesses associations of medical toxicology consultations with mortality outcomes among patients with poisonings who were admitted to the pediatric intensive care unit (PICU).

## Introduction

Pediatric toxicological exposures, including poisonings and overdoses, are a major cause of morbidity and mortality in children and adolescents.^1^ Many of these patients are cared for in a pediatric intensive care unit (PICU). At times, the treatment of these patients requires complex management decisions regarding antidote administration, airway support, choice of pressors, invasive physiologic interventions, and extracorporeal substance removal.^2,3^ Given the possibility of sudden cardiopulmonary deterioration after overdose, the PICU also provides an opportunity for close hemodynamic and respiratory monitoring even if the patient initially presents with stable vital signs. While some patients with toxicological exposures admitted to PICUs do not require invasive procedures and have a short length of stay (LOS), other toxicological exposures can be difficult to diagnose and may be associated with significant physiologic derangements warranting close monitoring for sudden decompensation.^4^ Pediatric poisonings (both intentional and unintentional substance or drug exposures) represent an estimated 3.1% to 8.0% of all PICU admissions.^5,6,7^

Medical toxicologists are physicians with subspecialty training in the pathophysiology, diagnosis, and treatment of patients with toxicological exposures. However, the number of medical toxicologists across the US is limited, and medical toxicologists are not universally staffed in hospitals. Medical toxicology bedside consultations have been associated with reduced mortality and LOS in a limited number of studies within single health systems^2,7^; however, it is unclear if this association exists specifically for patients who are admitted to the PICU. Furthermore, the association of medical toxicology consultations with mortality and LOS in any patient group has not been examined in a national setting. We hypothesized that consultations by medical toxicologists are associated with lower mortality and shorter LOS in poisoned patients requiring PICU care compared with cases without such consultations.

## Methods

This cross-sectional study was determined to be non–human participants research and to not require informed consent by the University of Texas Southwestern institutional review board and was conducted in accordance with the Declaration of Helsinki.^8^ The reporting of the study follows the Strengthening the Reporting of Observational Studies in Epidemiology (STROBE) reporting guideline.

Virtual Pediatric Systems (VPS) is a comprehensive database of 1.75 million cases of PICU admissions across 135 medical sites in the US. This database consists of deidentified information on demographics, clinical treatment, diagnoses, procedures, disposition, and outcome from the patient’s hospitalization. For this study, we selected only cases admitted to participating PICUs with a STAR diagnosis code for poisoning from January 1, 2019, to June 30, 2023. STAR codes are diagnosis categories created by VPS within their registry to designate diagnostics groupings. Readmissions of a given patient were excluded. Poisonings included toxicological exposures such as envenomations, intentional and unintentional overdoses, and adverse events related to medication errors. Documentation for bedside medical toxicology consultations were extracted from the electronic medical record by trained abstractors. Patients were classified as having a bedside medical toxicology consultation if one was documented in the VPS database. Poison center telephone consultations are not documented in VPS as receiving a medical toxicologist consultation.

### Variables

The primary outcomes consisted of the binary variables PICU mortality and hospital mortality. LOS variables, classified as medical, physical, and hospital, were all examined as secondary outcomes. Medical LOS refers to the time in the PICU that is medically necessary, excluding time spent waiting for transfer beds. The physical LOS is the total actual time the patient spent in the PICU. Hospital LOS is the entire duration of the patient’s hospital stay, including the PICU LOS. LOS was measured as the exact number of hours and minutes converted into a whole number of days (eg, 36 hours would be 1.5 days).

Demographic variables included age, sex, and race and ethnicity as obtained from the electronic medical record. Race and ethnicity categories included American Indian or Alaska Native, Asian, Black, Hispanic or Latino, Native Hawaiian or Other Pacific Islander, White, multiracial or other (defined as any race or ethnicity not otherwise specified), or unknown; race and ethnicity were included to account for disparities. Relevant covariates included trauma status, Pediatric Risk of Mortality version 3 (PRISM III),^9^ and Pediatric Index of Mortality version 3 (PIM3).^10^ Trauma status was operationalized as a binary variable (1 for yes and 0 for no). Patients were classified as having trauma if the principal cause of the patient’s injury requiring PICU admission was due to a physical trauma. PRISM III risk of mortality scores consist of 17 variables based on the clinical presentation, with the most predictive variables for mortality including systolic blood pressure, coma, and abnormal pupillary reflexes.^9^ The PRISM III risk of mortality score has been validated across multiple different pediatric diseases and conditions.^11,12,13^ Additionally, the PIM3 scores consist of 10 variables that are similar to the PRISM III risk of mortality score and adds diagnosis-related components.^10^ Both variables were included in the bivariate models and only the PRISM III risk of mortality score was used in the final multivariable models due to its superiority in discrimination capabilities.^11^

STAR codes and the *International Statistical Classification of Diseases and Related Health Problems, Tenth Revision (ICD-10)*^14^ were utilized to obtain poisoning exposure type. Four poisoning exposures were selected as control covariates based on their high severity: analgesics, antidepressants, cardiovascular agents, and opioids.

### Statistical Analysis

Patients who received a medical toxicology consultation were compared with those without consultations for the main analyses. Additionally, a subgroup analysis was conducted comparing those with medical toxicology consultations with those who had access to a medical toxicologist but did not receive a consultation. Age, sex, race and ethnicity, trauma, and PRISM III risk of mortality^10^ probability scores were examined between the 2 groups. Categorical data were compared using χ^2^ tests and Fisher exact tests. Continuous variables were examined for normality, and independent samples *t* tests or Wilcox Rank Sum tests were computed to ascertain statistically significant differences. Statistical significance was set at *P* < .05 a priori. Patients who had a LOS less than or equal to 30 days were included in the multivariable models, and all others with missing or longer LOS were excluded. All multivariable analyses were adjusted for age, sex, race and ethnicity, PRISM III risk of mortality score,^9^ and trauma status. Mixed-effect multilevel models were conducted to account for the clustering effects of hospital site characteristics. Multilevel models were computed for binary outcomes using a generalized linear model framework (PICU and hospital mortality) and continuous outcomes (medical, physical, and hospital LOS) using a γ distribution and log link function. The intercept for hospital was allowed to randomly vary across sites, and all other effects were fixed. For the multilevel models on LOS, PRISM III risk of mortality scores were scaled to represent the outcome corresponding to 1 SD difference in PRISM III risk of mortality score. Separate multivariable models were computed to account for selected exposure types. Model fit statistics were assessed, and all statistical assumptions were verified.

Odds ratios (ORs) and 95% CIs were computed for binary outcomes, and coefficient estimates and 95% CIs were computed for continuous outcomes. Log estimates were exponentiated to reflect changes in LOS outcomes for medical toxicology consultations. All variables were examined to ensure no more than 10% of cases were missing and that all data were assumed to be missing at random. Sensitivity analyses were conducted to examine models inclusive and exclusive of patients younger than 2 years. All analyses were conducted in R 4.2.2 (R Project for Statistical Computing) from May to August 2024.

## Results

Among the 52 836 PICU patients with poisoning diagnoses who represented 10% of the total PICU population in VPS from 2019 to 2023, 29 867 (56.5%) were adolescents and young adults (12 to <21 years), 29 401 (55.6%) were female, 7773 (14.7%) were Black, 4929 (14.7%) were Hispanic or Latino, and 27 324 (51.7%) were White (Table 1). A total of 2857 patients (5.4%) received medical toxicology consultations and 49 979 (94.6%) did not receive a consultation. Those who received medical toxicology consultations were slightly older, on average, compared with patients without consultations. Patients who received medical toxicology consultations had similar PRISM III mortality probabilities and PIM3 scores compared with those without consultations. However, the comparison group had a higher percentage of patients with trauma (2709 patients [5.4%]) compared with the medical toxicology consultation group (49 participants [1.7%]) (*P* < .001). Additionally, the percentage of mechanical ventilation was higher in the medical toxicology consultation group than the comparison group (16 193 participants [32.4%] vs 744 participants [26.0%]; *P* < .001). PICU mortality was lower for those with medical toxicology consultations compared with those without (22 participants [0.8%] vs 1020 participants [2.0%]; *P* < .001). Hospital mortality was also lower for those with medical toxicology consultations (28 participants [1.0%] vs 16 193 participants [2.6%]; *P* < .001). The median LOS (medical, physical, and hospital) was shorter for those who received a medical toxicology consultation compared with those without consultations.

**Table 1.** Descriptive Statistics of Overall Poisonings Among Patients Admitted to the PICU With or Without a Medical Toxicology Consultation

Characteristic	Participants No. (%)			χ^2^ (*df)*^a^	*P* value
	No medical toxicology consultation (n = 49 979)	Medical toxicology consultation (n = 2857)	Total (N = 52 836)		
Age, y					
12 to <21	27 873 (55.8)	1994 (69.8)	29 867 (56.5)	99.6 (3)	<.001
6 to <12	4622 (9.2)	140 (4.9)	4762 (9.0)		
2 to <6	7266 (14.5)	341 (11.9)	341 (11.9)		
0 to <2	10 218 (20.4)	382 (13.4)	382 (13.4)		
Sex at birth^b^					
Male	22 370 (44.8)	1062 (37.2)	23 432 (44.3)	62.8 (1)	<.001
Female	27 606 (55.2)	1795 (62.8)	29 401 (55.6)		
Missing or unknown	3 (0.1)	0	3 (0.1)		
Race and ethnicity					
American Indian or Alaska Native	722 (1.4)	42 (1.5)	764 (1.4)	111.8 (6)	<.001
Asian	1485 (3.0)	72 (2.5)	1557 (2.9)		
Black	7289 (14.6)	484 (16.9)	7773 (14.7)		
Hispanic or Latino	4749 (3.0)	180 (6.3)	4929 (9.3)		
Native Hawaiian or Other Pacific Islander	206 (0.4)	10 (0.4)	216 (0.4)		
White	25 643 (51.3)	1681 (58.8)	27 324 (51.7)		
Other or multiracial^c^	4641 (9.3)	155 (5.4)	4796 (9.1)		
Unknown	5244 (10.5)	233 (8.2)	5477 (10.4)		
PICU mortality	1020 (2.0)	22 (0.8)	1042 (2.0)	21.9 (1)	*<*.001
Hospital mortality	1270 (2.6)	28 (1.0)	1298 (2.5)	27.0 (1)	*<*.001
Mechanical ventilation in first 24 h	16 193 (32.4)	744 (26.0)	16 937 (32.1)	55.6 (1)	<.001
Trauma	2709 (5.4)	49 (1.7)	2758 (5.2)	74.21 (1)	<.001
PRISM III score					
0-4	37 363 (74.8)	2158 (75.5)	39 521 (74.8)	19.9 (4)	*<*.001
5-9	7890 (15.8)	495 (17.3)	8835 (15.9)		
10-14	2672 (5.3)	118 (4.1)	2790 (5.3)		
15-19	1055 (2.1)	45 (1.6)	1100 (2.1)		
>20	999 (2.0)	41 (1.4)	1040 (2.0)		
Probability of mortality					
PRISM III score, median (IQR)^d^	0.49 (0.34-1.14)	0.49 (0.34-1.14)	0.49 (0.34-1.14)	*W* = 70 739 866	.40
PIM 3 score, median (IQR)^e^	0.74 (0.65-2.79)	0.73 (0.69-1.57)	0.74 (0.65-2.79)	*W* = 70 860 536	.50
Length of stay, median (IQR), d					
Medical^f^	1.11 (0.64-2.83)	0.93 (0.58-1.57)	1.11 (0.64-2.71)	*W* = 78 425 181	<.001
Physical^g^	1.30 (0.76-3.12)	1.12 (0.73-1.77)	1.29 (0.76-2.98)	*W* = 80 983 065	<.001
Hospital^h^	2.71 (1.42-8.55)	2.46 (1.55-4.00)	2.69 (1.43-7.90)	*W* = 77 634 625	<.001
Analgesic exposures	3053 (6.1)	256 (9.0)	3309 (6.3)	37.0 (1)	<.001
Antidepressant exposures	1663 (3.3)	238 (8.3)	1901 (3.6)	193.6 (1)	<.001
Cardiovascular exposures	2368 (4.7)	224 (7.8)	2592 (4.9)	55.1 (1)	<.001
Opioid exposures	6251 (12.5)	213 (7.5)	6464 (12.2)	55.1 (1)	<.001
Medical length of stay, d^i^					
<1	22 534 (48.3)	1501 (54.1)	24 035 (48.6)	218.8 (4)	<.001
1.0-1.9	11 321 (24.2)	808 (29.1)	12 129 (24.5)		
2.0-2.9	3234 (6.9)	198 (7.1)	3432 (96.9)		
3.0- 6.9	3862 (8.3)	161 (5.8)	4023 (8.1)		
7.0-30	5747 (12.3)	109 (3.9)	5856 (11.6)		
Physical length of stay, d^i^					
<1	19 671 (41.2)	1274 (44.9)	20 945 (41.5)	227.0 (4)	<.001
1.0-1.9	13 447 (28.2)	995 (35.1)	14 442 (28.6)		
2.0-2.9	4059 (8.5)	251 (8.9)	4310 (8.5)		
3.0-6.9	4485 (9.4)	196 (6.9)	4681 (9.3)		
7.0-30	6027 (12.6)	120 (4.2)	6147 (12.2)		
Hospital length of stay, d^i^					
<1	8277 (18.7)	349 (12.5)	8626 (18.3)	356.2 (4)	<.001
1.0-1.9	11 661 (26.4)	789 (28.3)	12 450 (26.5)		
2.0-2.9	6666 (15.1)	647 (23.2)	7313 (15.6)		
3.0-6.9	9315 (21.1)	757 (27.1)	10 072 (21.4)		
7.0-30	8307 (18.8)	248 (8.9)	8555 (18.2)		

Abbreviations: PICU, pediatric intensive care unit; PIM 3, Pediatric Index of Mortality version 3; PRISM III, Pediatric Risk of Mortality version 3.

^a^ Continuous variables were tested using Wilcox Rank Sum tests (*W*).

^b^ Statistical testing did not include those with unknown or missing values.

^c^ Other includes any race or ethnicity not otherwise specified.

^d^ Range: 0.01 to 99.72.

^e^ Range: 0.02 to 99.89.

^f^ Range: 0.00 to 617.67 days.

^g^ Range: 0.01 to 617.67 days.

^h^ Range: 0.01 to 6579.80 days.

^i^ Excludes patients with lengths of stay greater than 30 days.

The multilevel model results (Table 2) for PICU and hospital mortality showed that medical toxicology consultations were associated with 69% lower odds of PICU mortality (adjusted OR [aOR], 0.31; 95% CI, 0.17 to 0.55) and 67% lower odds of hospital mortality (aOR, 0.33; 95% CI, 0.20 to 0.55), after adjusting for age, sex at birth, race and ethnicity, PRISM III risk of mortality score, and trauma. Mortality was also significantly associated with younger age, female sex (compared with male), higher PRISM III risk of mortality score, and trauma. Models for PICU and hospital mortality accounting for exposure type showed 64% lower odds of PICU mortality (aOR, 0.36; 95% CI, 0.20 to 0.63) and 61% lower odds of hospital mortality (aOR, 0.39; 95% CI, 0.24 to 0.64) for those who received a medical toxicology consultation, adjusting for all other covariates.

**Table 2.** Multilevel Model Results for Medical Toxicology Consultations and Mortality Among Patients Admitted to the PICU

Characteristic	Adjusted odds ratio (95% CI) (N = 47 359)^a^			
	PICU mortality	PICU mortality with poisoning types	Hospital mortality	Hospital mortality with poisoning types
Age (categorical)^b^	1.19 (1.12-1.27)	1.14 (1.07-1.22)	1.21 (1.15-1.28)	1.17 (1.10-1.24)
Sex at birth				
Male	1 [Reference]	1 [Reference]	1 [Reference]	1 [Reference]
Female	0.81 (0.69-0.95)	0.85 (0.73-0.99)	0.83 (0.72-0.95)	0.87 (0.76-1.00)
Race and ethnicity^c^				
Black	0.89 (0.71-1.17)	.89 (0.71-1.11)	0.98 (0.81-1.19)	0.98 (0.80-1.18)
Other	1.12 (0.93-1.34)	1.12 (0.93-1.34)	1.17 (0.99-1.38)	1.16 (0.99-1.37)
White	1 [Reference]	1 [Reference]	1 [Reference]	1 [Reference]
PRISM III probability of mortality	1.07 (1.07-1.08)	1.07 (1.07-1.08)	1.07 (1.07-1.08)	1.07 (1.05-1.10)
Received medical toxicology consultation	0.31 (0.17-0.55)	0.36 (0.20-0.63)	0.33 (0.20-0.55)	0.39 (0.24-0.64)
Trauma	2.28 (1.78-2.92)	2.21 (1.73-2.84)	1.76 (1.39-2.25)	1.69 (1.33-2.15)
Analgesic poisoning	NA	0.52 (0.30-0.90)	NA	0.40 (0.23-0.68)
Antidepressant poisoning	NA	0.26 (0.11-0.63)	NA	0.20 (0.08-0.48)
Cardiovascular agent poisoning	NA	0.61 (0.35-1.06)	NA	0.48 (0.28-0.81)
Opioid poisoning	NA	1.31 (1.06-1.63)	NA	1.19 (0.98-1.44)

Abbreviations: NA, not applicable; PICU, pediatric intensive care unit; PRISM III, Pediatric Risk of Mortality version 3.

^a^ These models include a random intercept for hospital and fixed effects for level 1 variables. Models were adjusted for age, sex at birth, race and ethnicity, PRISM III, and trauma.

^b^ Age is an ordinal variable with the following mutually exclusive groups in descending order: 12 years to younger than 21 years, 6 years to younger than 12 years, and 0 to younger than 2 years. The reference group for age was 12 years to younger than 21 years.

^c^ Race and ethnicity was a combined variable until 2021, and then they were reported using the Office of Management and Budget standards. Prior to 2021, Hispanic ethnicity was measured in the combined race and ethnicity variable. Other race and ethnicity includes American Indian or Alaska Native, Asian, Hispanic or Latino, Native Hawaiian or Other Pacific Islander, and multiracial. Regression models excluded participants where race and ethnicity was unknown (5477 individuals).

Multilevel log-linear regression results are displayed in Table 3. After controlling for clustering effects within hospitals and other covariates, medical toxicology consultations were associated with a 31% reduction in medical LOS (log estimate, −0.37; 95% CI, −0.42 to −0.32), a 30% reduction in physical LOS (log estimate, −0.36; 95% CI, −0.40 to −0.31), and a 25% reduction in hospital LOS (log estimate, −0.29; 95% CI, −0.33 to −0.25). When adjusting for exposure type, those who received medical toxicology consultations had a 15% reduction in medical LOS (log estimate, −0.16; 95% CI, −0.21 to −0.11) and physical LOS (log estimate, −0.16; 95% CI, −0.21 to −0.12) and a 10% reduction in hospital LOS (log estimate, −0.10; 95% CI, −0.14 to −0.06). Sensitivity analyses showed similar findings for the association of medical toxicology consultations with outcomes when excluding patients younger than 2 years of age.

**Table 3.** Multilevel Regression Results for Medical, Physical, and Hospital Length of Stay Among Poisoned Patients in the Pediatric Intensive Care Unit

Characteristic	Adjusted log estimate (95% CI) (N = 45 191)^a^					
	Medical length of stay		Physical length of stay		Hospital length of stay	
	Model 1	Model 2	Model 1	Model 2	Model 1	Model 2
Age (categorical)^b^	0.29 (0.28 to 0.29)	0.16 (0.15 to 0.17)	0.26 (0.25 to 0.27)	0.14 (0.13 to 0.14)	0.13 (0.12 to 0.14)	0.10 (0.09 to 0.11)
Sex at birth						
Male	1 [Reference]	1 [Reference]	1 [Reference]	1 [Reference]	1 [Reference]	1 [Reference]
Female	−0.11 (−0.13 to −0.09)	0.01 (0.01 to 0.01)	−0.09 (−0.11 to −0.07)	0.01 (−0.01 to 0.01)	−0.03 (−0.04 to 0.01)	0.01 (−0.01 to 0.01)
Race and ethnicity^c^						
Black	−0.02 (−0.05 to 0.01)	−0.04 (0.07 to −0.01)	−0.03 (−0.06 to 0.01)	−0.04 (−0.07 to −0.02)	−0.01 (−0.04 to 0.01)	−0.05 (−0.07 to −0.02)
Other	0.01 (−0.02 to 0.03)	0.02 (−0.01 to 0.04)	0.12 (0.10 to 0.14)	0.02 (−0.01 to 0.04)	0.02 (−0.01 to 0.04)	0.01 (−0.02 to 0.02)
White	1 [Reference]	1 [Reference]	1 [Reference]	1 [Reference]	1 [Reference]	1 [Reference]
PRISM III mortality score^d^	0.30 (0.29 to 0.32)	0.02 (0.02 to 0.02)	0.29 (0.27 to 0.30)	0.02 (0.02 to 0.02)	0.18 (0.17 to 0.19)	0.02 (0.02 to 0.02)
Received medical toxicology consultation	−0.37 (−0.42 to −0.32)	−0.16 (−0.21 to −0.11)	−0.36 (−0.40 to −0.31)	−0.16 (−0.21 to −0.12)	−0.29 (−0.33 to −0.25)	−0.10 (−0.14 to −0.06)
Trauma	0.41 (0.37 to 0.45)	0.30 (0.25 to 0.34)	0.38 (0.34 to 0.42)	0.28 (0.24 to 0.32)	0.37 (0.33 to 0.41)	0.20 (0.16 to 0.24)
Analgesics	NA	−0.11 (−0.15 to −0.07)	NA	−0.07 (−0.11 to −0.03)	NA	−0.04 (−0.08 to −0.01)
Antidepressants	NA	0.01 (−0.05 to 0.05)	NA	0.01 (−0.04 to 0.05)	NA	0.04 (−0.01 to 0.08)
Cardiovascular agents	NA	−0.36 (−0.41 to −0.32)	NA	−0.36 (−0.40 to −0.32)	NA	−0.30 (−0.34 to −0.26)
Opioids	NA	0.70 (0.66 to 0.73)	NA	0.65 (0.62 to 0.68)	NA	0.57 (0.54 to 0.60)

Abbreviations: NA, not applicable; PRISM III, Pediatric Risk of Mortality version 3.

^a^ Regression models included a log link function for a γ distribution. These models excluded participants where race and ethnicity was unknown (5477 individuals) and length of stay was unknown (medical, 1144 individuals; hospital, 276 individuals). These models also excluded those who had a length of stay greater than 30 days (medical, 2217 individuals; physical, 2311 individuals; hospital, 5544 individuals). These models include a random intercept for hospital and fixed effects for level 1 variables. Model 1 was adjusted for age, sex at birth, race and ethnicity, PRISM III, and trauma. Model 2 was adjusted for all covariates listed in model 1 in addition to selected poisoning types.

^b^ Age is a categorical variable with the following mutually exclusive groups: 12 years to younger than 21 years, 6 years to younger than 12 years, and 0 to younger than 2 years. The reference group for age was 12 years to younger than 21 years.

^c^ Race and ethnicity was a combined variable until 2021, and then they were reported using the Office of Management and Budget standards. Prior to 2021, Hispanic ethnicity was measured in the combined race and ethnicity variable. Other race and ethnicity includes American Indian or Alaska Native, Asian, Hispanic or Latino, Native Hawaiian or Other Pacific Islander, and multiracial.

^d^ PRISM III mortality probability was scaled for model fit to represent the change in length of stay for 1 SD difference in mortality probability scores.

The subgroup analysis comparing those who had a medical toxicology consultation with those who did not receive a consultation but had access to a medical toxicologist yielded a total of 14 783 patients (Table 4). After adjusting for covariates and exposure type, medical toxicology consultations were associated with 70% lower odds of PICU mortality (aOR, 0.30; 95% CI, 0.17 to 0.56) and 66% lower odds of hospital mortality (aOR, 0.34; 95% CI, 0.20 to 0.58). Medical toxicology consultations were also associated with a 14% shorter LOS for all 3 types (medical: log estimate, −0.14; 95% CI, −0.19 to −0.08; physical: log estimate, −0.14; 95% CI, −0.19 to −0.09; hospital: log estimate, −0.14; 95% CI, −0.19 to −0.09), after adjusting for covariates and exposure type (Table 5).

**Table 4.** Multilevel Model Results for Medical Toxicology Consultations and Mortality Among Patients Admitted to the PICU With Access to Medical Toxicologists

Characteristic	Adjusted odds ratio (95% CI) (N = 14 783)^a^			
	PICU mortality	PICU mortality with poisoning types	Hospital mortality	Hospital mortality with poisoning types
Age (categorical)^b^	1.19 (1.12-1.27)	1.15 (1.02-1.29)	1.21 (1.15-1.28)	1.13 (1.02-1.25)
Sex at birth				
Male	1 [Reference]	1 [Reference]	1 [Reference]	1 [Reference]
Female	0.81 (0.69-0.95)	0.91 (0.69-1.19)	0.83 (0.72-0.95)	0.95 (0.75-1.21)
Race and ethnicity^c^				
Black	0.89 (0.71-1.17)	0.61 (0.40-0.93)	0.98 (0.81-1.19)	0.72 (0.50-1.02)
Other	1.12 (0.93-1.34)	0.99 (0.71-1.38)	1.17 (0.99-1.38)	0.95 (0.71-1.27)
White	1 [Reference]	1 [Reference]	1 [Reference]	1 [Reference]
PRISM III probability of mortality	1.07 (1.07-1.08)	1.07 (1.07-1.08)	1.07 (1.07-1.08)	1.07 (1.07-1.08)
Received medical toxicology consultation	0.31 (0.17-0.55)	0.30 (0.17-0.56)	0.33 (0.20-0.55)	0.34 (0.20-0.58)
Trauma	2.28 (1.78-2.92)	2.32 (1.63-3.52)	1.76 (1.39-2.25)	1.60 (1.06-2.41)
Analgesic poisoning	NA	0.56 (0.18-1.79)	NA	0.37 (0.12-1.15)
Antidepressant poisoning	NA	0.21 (0.05-1.00)	NA	0.14 (0.02-0.66)
Cardiovascular agent poisoning	NA	0.93 (0.37-2.39)	NA	0.64 (0.25-1.60)
Opioid poisoning	NA	1.44 (1.01-2.05)	NA	1.41 (1.04-1.92)

Abbreviations: NA, not applicable; PICU, pediatric intensive care unit; PRISM III, Pediatric Risk of Mortality version 3.

^a^ Regression models excluded participants where race and ethnicity was unknown (1931 individuals). These models include a random intercept for hospital and fixed effects for level 1 variables. Models were adjusted for age, sex at birth, race and ethnicity, PRISM III, and trauma.

^b^ Age is an ordinal variable with the following mutually exclusive groups in descending order: 12 years to less than 21 years, 6 years to less than 12 years, and 0 to less than 2 years. The reference group for age was 12 years to less than 21 years.

^c^ Race and ethnicity was a combined variable until 2021, and then they were reported using the Office of Management and Budget standards. Prior to 2021, Hispanic ethnicity was measured in the combined race and ethnicity variable. Other race and ethnicity includes American Indian or Alaska Native, Asian, Hispanic or Latino, Native Hawaiian or Other Pacific Islander, and multiracial.

**Table 5.** Multilevel Regression Results for Medical, Physical, and Hospital Length of Stay Among Poisoned Patients in the Pediatric Intensive Care Unit With Access to Medical Toxicologists

Characteristic	Adjusted log estimate (95% CI) (N = 14 783)^a^					
	Medical length of stay		Physical length of stay		Hospital length of stay	
	Model 1	Model 2	Model 1	Model 2	Model 1	Model 2
Age (categorical)^b^	0.27 (0.26 to 0.29)	0.18 (0.16 to 0.19)	0.25 (0.24 to 0.26)	0.16 (0.14 to 0.17)	0.12 (0.10 to 0.13)	0.05 (0.03 to 0.07)
Sex at birth						
Male	1 [Reference]	1 [Reference]	1 [Reference]	1 [Reference]	1 [Reference]	1 [Reference]
Female	−0.08 (−0.12 to −0.04)	−0.05 (−0.08 to −0.01)	−0.06 (−0.09 to −0.02)	−0.03 (−0.06 to 0.01)	0.01 (−0.03 to 0.05)	0.03 (−0.01 to 0.06)
Race and ethnicity^c^						
Black	−0.10 (−0.16 to −0.05)	−0.13 (−0.19 to −0.08)	−0.10 (−0.15 to −0.05)	−0.13 (−0.18 to −0.08)	−0.10 (−0.15 to −0.05)	−0.09 (−0.14 to −0.04)
Other	0.03 (−0.02 to 0.08)	−0.03 (−0.08 to 0.01)	0.03 (−0.02 to 0.07)	−0.03 (−0.08 to 0.01)	−0.01 (−0.05 to 0.04)	−0.04 (−0.09 to 0.01)
White	1 [Reference]	1 [Reference]	1 [Reference]	1 [Reference]	1 [Reference]	1 [Reference]
PRISM III mortality score^d^	0.22 (0.22 to 0.22)	0.02 (0.02 to 0.02)	0.02 (0.02 to 0.02)	0.02 (0.02 to 0.02)	0.01 (0.01 to 0.01)	0.01 (0.01 to 0.01)
Received medical toxicology consultation	−0.29 (−0.34 to −0.24)	−0.14 (−0.19 to −0.08)	−0.28 (−0.33 to −0.24)	−0.14 (−0.19 to −0.09)	−0.22 (−0.26 to −0.17)	−0.14 (−0.19 to −0.09)
Trauma	0.29 (0.20 to 0.37)	0.33 (0.25 to 0.41)	0.27 (−0.19 to 0.35)	0.30 (0.23 to 0.38)	0.28 (0.19 to 0.36)	0.29 (0.21 to 0.36)
Analgesics	NA	−0.25 (−0.34 to −0.16)	NA	−0.20 (−0.28 to −0.12)	0.12 (0.10 to 0.13)	0.10 (0.02 to 0.18)
Antidepressants	NA	0.01 (−0.09 to 0.09)	NA	0.01 (−0.08 to 0.10)	NA	0.05 (−0.03 to 0.14)
Cardiovascular agents	NA	−0.37 (−0.46 to −0.29)	NA	−0.37 (−0.45 to −0.30)	NA	−0.45 (−0.52 to −0.37)
Opioids	NA	0.83 (0.77 to 0.89)	NA	0.77 (0.72 to 0.82)	NA	0.68 (0.63 to 0.74)

Abbreviations: NA, not applicable; PRISM III, Pediatric Risk of Mortality version 3.

^a^ Regression models included a log link function for a γ distribution. These models excluded participants where race and ethnicity was unknown (1931 individuals) and length of stay was unknown (medical, 244 individuals; hospital, 150 individuals). These models also exclude those who had a length of stay greater than 30 days (medical, 683 individuals; physical, 712 individuals; hospital, 1786 individuals). These models include a random intercept for hospital and fixed effects for level 1 variables. Model 1 was adjusted for age, sex at birth, race and ethnicity, PRISM III, and trauma. Model 2 was adjusted for all covariates listed in model 1 in addition to selected poisoning types.

^b^ Age is a categorical variable with the following mutually exclusive groups: 12 years to younger than 21 years, 6 years to younger than 12 years, 0 to younger than 2 years. The reference group for age was 12 years to younger than 21 years.

^c^ Race and ethnicity was a combined variable until 2021, and then they were reported using the Office of Management and Budget standards. Prior to 2021, Hispanic ethnicity was measured in the combined race and ethnicity variable. Other race and ethnicity includes American Indian or Alaska Native, Asian, Hispanic or Latino, Native Hawaiian or Other Pacific Islander, and multiracial.

^d^ PRISM III mortality probability was scaled for model fit to represent the change in length of stay for 1 SD difference in mortality probability scores.

## Discussion

This cross-sectional study is the first, to our knowledge, to examine associations of medical toxicology consultations with mortality and LOS outcomes among poisoned patients admitted to the PICU in a national setting. Utilizing a large dataset consisting of multiple hospitals, this study found that receiving medical toxicology consultations was associated with reduced odds of mortality and LOS, even after adjusting for patient severity, trauma, age, sex at birth, race and ethnicity, selected exposure types, and the effects attributed to specific hospital sites.

Limited studies have examined associations of medical toxicology consultations with LOS and mortality, but these investigations were restricted to single health care systems. Additionally, these studies included both adults and children who were treated in all areas of the hospital.^2,7^ The current study fills an important gap by focusing on PICU patients specifically. Pediatric patients who require PICU stays are often particularly vulnerable to adverse health outcomes related to toxicological exposures, including rapid deterioration after ingestions. Additionally, pediatric patients are usually unable to recall what they were exposed to, particularly for exploratory ingestions for those younger than 2 years. These patients are difficult to diagnose and treat because the clinician often relies on parental recall and clinical signs and symptoms of the exposed patient. The current study found that medical toxicology consultations were associated with clinically significant reductions in mortality and LOS, emphasizing the importance of bedside medical toxicology consultations for PICU patients with toxicological exposures.

Identifying the mechanisms underlying the association of medical toxicology consultations with PICU mortality was outside the scope of this study and was restricted to variables available in the VPS database. However, previous research has shown that medical toxicologists are more likely to quickly identify toxidromes, rapidly identify pharmaceuticals for antidote administration, and anticipate potential complications from poisonings that may arise after the patient is admitted to the PICU.^2^ Future studies should examine the specific factors (eg, procedures, medication administration, and patient complications in the ICU) that may explain the association of medical toxicology consultation with reduction in mortality and LOS.

The mortality of poisoned PICU patients in our study (2.0%) was similar to prior research (1.9%).^15^ One study,^5^ which examined PICU interventions for poisoned patients (also using the VPS dataset), found that the majority of poisoned patients did not require procedures necessitating ICU admission. In fact, most poisoned PICU patients received only continuous monitoring, which was the reason for the ICU stay.^5^

### Limitations

While this study is the first, to our knowledge, to examine the association of medical toxicology consultations with mortality and LOS in the PICU, several limitations should be noted. First, we did not examine procedures in this specific analysis, which may help to explain the associations of medical toxicology consultations with mortality. A future analysis should evaluate procedures used in patients who received medical toxicology consultations. Additionally, specifics about antidotal therapy and pharmacological interventions were not available in the VPS dataset. In January 2021, VPS standardized race and ethnicity to the US Census Bureau and the Office of Management and Budget standards. However, prior to this, race and ethnicity were operationalized as a single measure, and not all sites collected this information. As such, patients with unknown race and ethnicity were excluded from the multivariable models. Future studies should replicate these analyses with more recent VPS data incorporating Office of Management and Budget standard classifications. While medical toxicology consultations are documented in VPS, the absence of medical toxicology consultations at sites with access to a medical toxicologist may not be comprehensive. Therefore, the comparison group contains those who do not have access to medical toxicologists and those who have access but did not receive a consultation. There also may be patients who were misclassified as not having a medical toxicologist consultation when in fact they did receive one; however, we expect this misclassification bias to be minimal. In the current study, our analyses accounted for severity of patients’ conditions using the PRISM III risk of mortality score. However, it is unclear if the PRISM III risk of mortality score adequately predicts mortality or severity of illness in poisonings because they represent a group of physiologically diverse disorders.^16^ Additionally, the dataset did not have information on the timing of receiving a medical toxicology consultation, which might be important in understanding the association with mortality and LOS.

## Conclusions

This cross-sectional study found that medical toxicology consultations were associated with lower mortality and shorter LOS among poisoned patients in the VPS dataset. Medical toxicology consultations may provide life-saving treatment, particularly for the most severe poisonings in the PICU. Future studies should examine larger subgroups of exposure types and types of procedures for poisoned patients to further understand the mechanisms through which medical toxicology consultations are associated with mortality in the PICU specifically.

## Notes

Supplement.
Data Sharing Statement

## References

[1] Gaw CE, Curry AE, Osterhoudt KC, Wood JN, Corwin DJ. Characteristics of fatal poisonings among infants and young children in the United States. Pediatrics. 2023;151(4):e2022059016. doi:10.1542/peds.2022-059016 36897244

[2] Curry SC, Brooks DE, Skolnik AB, Gerkin RD, Glenn S. Effect of a medical toxicology admitting service on length of stay, cost, and mortality among inpatients discharged with poisoning-related diagnoses. J Med Toxicol. 2015;11(1):65-72. doi:10.1007/s13181-014-0418-z 25127915 PMC4371032

[3] Love JS, Karshenas DL, Spyres MB, ; Toxicology Investigators Consortium Study Group. The toxicology investigators consortium case registry—the 2021 annual report. J Med Toxicol. 2022;18(4):267-296. doi:10.1007/s13181-022-00910-6 36070069 PMC9450833

[4] Even KM, Armsby CC, Bateman ST. Poisonings requiring admission to the pediatric intensive care unit: a 5-year review. Clin Toxicol (Phila). 2014;52(5):519-524. doi:10.3109/15563650.2014.909601 24738737

[5] Patel MM, Travers CD, Stockwell JA, Geller RJ, Kamat PP, Grunwell JR. Analysis of interventions required in 12,021 children with acute intoxications admitted to PICUs. Pediatr Crit Care Med. 2017;18(7):e281-e289. doi:10.1097/PCC.0000000000001187 28481828 PMC6310173

[6] Lacroix J, Gaudreault P, Gauthier M. Admission to a pediatric intensive care unit for poisoning: a review of 105 cases. Crit Care Med. 1989;17(8):748-750. doi:10.1097/00003246-198908000-00005 2486553

[7] King AM, Danagoulian S, Lynch M, . The effect of a medical toxicology inpatient service in an academic tertiary care referral center. J Med Toxicol. 2019;15(1):12-21. doi:10.1007/s13181-018-0684-2 30353414 PMC6314930

[8] World Medical Association. World Medical Association Declaration of Helsinki: ethical principles for medical research involving human subjects. JAMA. 2013;310(20):2191-2194. doi:10.1001/jama.2013.281053 24141714

[9] Pollack MM, Patel KM, Ruttimann UE. PRISM III: an updated pediatric risk of mortality score. Crit Care Med. 1996;24(5):743-752. doi:10.1097/00003246-199605000-00004 8706448

[10] Straney L, Clements A, Parslow RC, ; ANZICS Paediatric Study Group and the Paediatric Intensive Care Audit Network. Pediatric index of mortality 3: an updated model for predicting mortality in pediatric intensive care*. Pediatr Crit Care Med. 2013;14(7):673-681. doi:10.1097/PCC.0b013e31829760cf 23863821

[11] Rahmatinejad Z, Rahmatinejad F, Sezavar M, Tohidinezhad F, Abu-Hanna A, Eslami S. Internal validation and evaluation of the predictive performance of models based on the PRISM-3 (Pediatric Risk of Mortality) and PIM-3 (Pediatric Index of Mortality) scoring systems for predicting mortality in pediatric intensive care units (PICUs). BMC Pediatr. 2022;22(1):199. doi:10.1186/s12887-022-03228-y 35413854 PMC9004120

[12] Shen Y, Jiang J. Meta-analysis for the prediction of mortality rates in a pediatric intensive care unit using different scores: PRISM-III/IV, PIM-3, and PELOD-2. Front Pediatr. 2021;9:712276. doi:10.3389/fped.2021.712276 34504815 PMC8421854

[13] Taori RN, Lahiri KR, Tullu MS. Performance of PRISM (pediatric risk of mortality) score and PIM (pediatric index of mortality) score in a tertiary care pediatric ICU. Indian J Pediatr. 2010;77(3):267-271. doi:10.1007/s12098-010-0031-3 20177831

[14] World Health Organization. International Classification of Diseases and Related Health Problems (10th Rev.). World Health Organization; 2016.

[15] Ozdemir R, Bayrakci B, Tekşam O, Yalçin B, Kale G. Thirty-three-year experience on childhood poisoning. Turk J Pediatr. 2012;54(3):251-259. 23094535

[16] Schwarz ES, Kopec KT, Wiegand TJ, Wax PM, Brent J. Should we be using the poisoning severity score? J Med Toxicol. 2017;13(2):135-145. doi:10.1007/s13181-017-0609-5 28283941 PMC5440322

