# Supplementary material for: Medical Toxicology Consultations and Mortality Among Patients With Poisonings in the PICU
Source: JAMA Netw Open. 2025 Feb 28;8(2):e2462139. doi: 10.1001/jamanetworkopen.2024.62139 (PMC11871539; doi:10.1001/jamanetworkopen.2024.62139)
Supplement: Supplement. — Data Sharing Statement [file jamanetwopen-e2462139-s001.pdf]

# Data Sharing Statement

Wax. Medical Toxicology Consultations and Mortality Among Children, Adolescents, and Young Adults With Poisonings. *JAMA Netw Open*. Published February 28, 2025. doi:10.1001/jamanetworkopen.2024.62139

**Data**
**Data available:** No
